# Supplementary material for: Short- and Long-Term Outcomes of Patients with Postoperative Arrhythmia after Liver Surgery
Source: Biomedicines. 2024 Jan 25;12(2):271. doi: 10.3390/biomedicines12020271 (PMC10886928; doi:10.3390/biomedicines12020271)
Supplement: Supplementary file 1 [file biomedicines-12-00271-s001.zip › Figure S1.pdf]

Universitätsmedizin Göttingen
Allgemein-, Viszeral- und Kinderchirurgie, Univ.-Prof. Dr. med. Michael Ghadimi
Robert-Koch-Str. 40, 37075 Göttingen

UNIVERSITÄTSMEDIZIN GÖTTINGEN : UMG
Klinik für Allgemein-, Viszeral- und Kinderchirurgie
Direktor: Univ.-Prof. Dr. med. Michael Ghadimi

Questionnaire

Please check the appropriate items!

37099 Göttingen **Briefpost**
Robert-Koch-Straße 40, 37075 Göttingen **Adresse**
0551 39-66104 **Telefon**
0551 39-12550 **Fax**
e.deborahchristina@stud.uni-goettingen.de **E-Mail**

Göttingen, (date)

Patient data

Patient's name (*birthdate)
Kind of liver surgery: name (date)

1) Were you aware of a cardiac arrhythmia (e.g. atrial fibrillation) before the liver operation?

Yes (if so, what kind of arrhythmia was it?)		No

2) Have you developed a permanent or recurring cardiac arrhythmia in the period after the operation?

Yes (if so, what kind of arrhythmia was diagnosed?)		No

If true, in what period after the operation did the cardiac arrhythmia occur?

In hospital	In the first 4 weeks after discharge	In the first 3 months after discharge	In the first 6 months after discharge	In the first year after discharge	Later than 1 year after discharge

3) Have you suffered a stroke in the period after the operation?

Yes		No

If true, in what period after the operation did the stroke occur?

In hospital	In the first 4 weeks after discharge	In the first 3 months after discharge	In the first 6 months after discharge	In the first year after discharge	Later than 1 year after discharge

4) Do you take blood-thinning medication (e.g. ASS, Marcumar, Eliquis etc.)?

Yes (if so, which ones and since when or how long?)		No

If true, why do you take this medication?

Arrhythmia	Cardiovascular disease	Thrombosis	Embolic event	Other:

5) Did you have any heart surgery in the period after the surgery?

Yes (if so, when exactly?)		No

If so, why (what was the underlying diagnosis)?

Universitätsmedizin Göttingen, Georg-August-Universität Stiftung Öffentlichen Rechts **Vorstand** Prof. Dr. Heyo K. Kroemer (Forschung & Lehre, Sprecher des Vorstands) Dr. Martin Siess (Krankenversorgung) Dr. Sebastian Freytag (Wirtschaftsführung & Administration) **Sparkasse Göttingen** (260 500 01) Kto: 448
